# Association of Proton Pump Inhibitor and Potassium‐Competitive Acid Blocker Use With Discontinuation and Intolerance of Oral 5‐Aminosalicylic Acid in Patients With Ulcerative Colitis

**DOI:** 10.1002/jgh3.70350

**Published:** 2026-01-31

**Authors:** Shinsuke Otagiri, Takahiro Ito, Keiji Yagisawa, Ayumu Sugitani, Atsuo Maemoto

**Affiliations:** ^1^ Inflammatory Bowel Disease Center Sapporo Higashi Tokushukai Hospital Sapporo Hokkaido Japan

**Keywords:** 5‐aminosalicylic acid, proton pump inhibitors, ulcerative colitis

## Abstract

**Aims:**

Proton pump inhibitors (PPIs) and potassium‐competitive acid blockers (PCABs) are widely used for acid‐related disorders. Recent studies have raised concerns that acid suppression may alter gut microbiota and drug pharmacokinetics, potentially influencing therapeutic outcomes in patients with inflammatory bowel disease. However, the impact of PPI/PCAB use on oral 5‐aminosalicylic acid (5‐ASA) therapy and intolerance in ulcerative colitis (UC) remains unclear. We aimed to examine whether concomitant PPI/PCAB use was associated with 5‐ASA discontinuation and intolerance in patients with UC.

**Methods:**

We retrospectively analyzed consecutive patients who received oral 5‐ASA for the first time between 2015 and 2022 at a single tertiary center. Patients receiving concomitant steroids or cytapheresis at baseline were excluded. The primary outcome was the association between PPI/PCAB use and 5‐ASA intolerance, and the secondary outcome was the association with treatment discontinuation.

**Results:**

A total of 181 patients were included in this study. The cumulative continuation rates of oral 5‐ASA at 1, 3, and 5 years were 60.4%, 45.4%, and 39.3%, respectively. Overall, 29 patients (16.0%) developed 5‐ASA intolerance. In multivariate analyses, concomitant PPI/PCAB use was independently associated with 5‐ASA intolerance (OR 9.65, 95% CI 1.92–48.5, *p* < 0.01) and treatment discontinuation (HR 2.46, 95% CI 1.06–5.65, *p* = 0.034), whereas age ≥ 40 years was associated with lower odds of intolerance.

**Conclusion:**

Concomitant PPI/PCAB use was associated with higher rates of 5‐ASA discontinuation and intolerance in patients with UC. These findings suggest novel associations and warrant validation in larger, prospective cohorts.

## 1 Introduction

Ulcerative colitis (UC) is a chronic inflammatory disorder that mainly affects the colon. Although the mechanism of UC remains unclear, intestinal inflammation occurs because of an overactive immune response and imbalanced intestinal microbiota [1]. Although therapeutic options for UC have expanded in recent decades [2, 3], 5‐aminosalicylic acid (5‐ASA) remains the first‐line therapy for mild to moderate UC [4]. By contrast, the incidence of 5‐ASA intolerance has been reported to be up to 16.2% between 2014 and 2016 in Japan [5]. 5‐ASA intolerance typically occurs within weeks of initiation and presents with systemic and gastrointestinal symptoms [6]. The mechanisms remain unclear, and 5‐ASA intolerance has been associated with intestinal microbiota dysbiosis [7, 8].

Proton pump inhibitors (PPIs) are the most widely used agents for the suppression of gastric acid [9]. Potassium‐competitive acid blockers (PCABs) are a newer class of acid‐suppressing agents that inhibit the gastric H+/K+‐ATPase by competitively blocking potassium binding. Compared with PPIs, PCABs have a more rapid onset of action and more potent acid suppression [10]. PCABs have been increasingly used for acid‐related disorders, particularly in Japan [11].

PPIs and PCABs can alter the gut microbiota [12, 13]. PPIs have been associated with dysbiosis through direct effects on bacterial proton pumps and acid suppression–induced changes in the gut environment [14]. Long‐term PPI use reduces gut butyrate levels in animal models, suggesting impaired short‐chain fatty acid production and dysbiosis [15]. PPI‐induced dysbiosis may impair intestinal barrier function [16] and promote pro‐inflammatory bacterial overgrowth and immune hyperreactivity [17], potentially increasing the risk of 5‐ASA intolerance.

Regular PPI use has been associated with an increased risk of IBD and worse clinical outcomes [18, 19]; however, the effects of PPI or PCAB on 5‐ASA treatment or 5‐ASA intolerance in UC are still unknown. Only one retrospective study has examined oral mesalamine with or without PPI and suggested that PPI use may not significantly alter the efficacy of mesalamine in active UC. However, the findings are limited by the observational design and should be interpreted in the context of its methodological constraints [20].

Thus, the objective of this study was to investigate whether the use of PPIs or PCABs is associated with oral 5‐ASA treatment discontinuation and/or intolerance in patients with UC.

## 2 Methods

### 2.1 Study Design

This was a single‐center, retrospective, observational study. The observation period was from January 2015 to November 2024.

### 2.2 Participants

The inclusion criteria:

1. Patients who were diagnosed with UC in accordance with the Japanese diagnostic criteria for UC [21], based on a combination of clinical history, endoscopic findings, and histologic evidence, along with the exclusion of infectious and other differential diagnoses. Alternative diagnoses were ruled out by stool cultures, *Clostridioides difficile* toxin testing, and appropriate imaging. Patients with a partial Mayo score of 0 at baseline were regarded as UC if they had a documented history of typical symptoms or reproducible endoscopic and histologic findings.
2. Patients with UC who received the first oral 5‐ASA administration between January 2015 and December 2022 at our hospital. Patients who had received any 5‐ASA formulation before referral were excluded.
3. Patients who underwent at least one follow‐up after starting 5‐ASA administration.

The exclusion criteria:

1. Patients treated exclusively with topical (enema or suppositories) 5‐ASA were excluded from the main cohort but were evaluated separately as a supplementary analysis.
2. Patients receiving concomitant steroid therapy (including topical formulations) or cytapheresis at initial 5‐ASA administration were excluded to isolate the effects of 5‐ASA monotherapy and avoid confounding that could mask or modify intolerance symptoms.

### 2.3 Outcomes

The primary outcome was the association between PPI/PCAB use and 5‐ASA intolerance.

A secondary outcome was the association between PPI/PCAB use and 5‐ASA treatment discontinuation.

### 2.4 Definitions

Patients were classified into the PPI/PCAB group if they were receiving a PPI or PCAB at initiation of oral 5‐ASA therapy; all others were assigned to the non‐PPI/PCAB group. Exposure was defined at baseline only, and subsequent changes during follow‐up were not considered.

The 5‐ASA continuation rate was defined as persistence of the initial oral formulation, and Kaplan–Meier analysis was restricted to this initial treatment period.

5‐ASA discontinuation was defined as withdrawal of or switching from the initial oral 5‐ASA due to intolerance or inadequate response. Switching formulations because of insufficient efficacy was considered discontinuation of the initial therapy.

Patients who discontinued 5‐ASA because of transfer to another hospital, self‐discontinuation without documented intolerance or inefficacy, or patient preference were censored at the last follow‐up.

Regarding the reasons for 5‐ASA discontinuation, primary nonresponse was defined as the absence of clinical improvement within 12 weeks of initiating oral 5‐ASA. Secondary nonresponse was defined as loss of efficacy after an initial response. Endoscopic nonresponse was defined as a lack of mucosal healing, represented by a Mayo endoscopic subscore of ≥ 2 at follow‐up colonoscopy. 5‐ASA intolerance was analyzed separately as the primary outcome.

Escalation of the initial oral 5‐ASA dose or the addition of topical 5‐ASA or other UC treatments (such as corticosteroids, cytapheresis, thiopurine, or biologics) was not considered discontinuation as long as the initial oral 5‐ASA formulation was maintained.

5‐ASA intolerance was defined as development of characteristic symptoms (fever, headache, skin rash, hepatic dysfunction, pancreatitis, pneumonitis, or worsened gastrointestinal symptoms such as nausea, abdominal pain, or diarrhea) within 4 weeks of treatment initiation, with resolution of symptoms within 1 week after drug withdrawal (temporal criteria). Patients fulfilling the temporal criteria were classified as intolerant without rechallenge. When rechallenge was attempted, intolerance was confirmed if symptoms recurred, whereas tolerance upon rechallenge was considered evidence against intolerance. In patients who did not fully meet the temporal criteria, the diagnosis was only established when supported by objective confirmation, including a positive drug lymphocyte stimulation test (DLST) or reproducibility of symptoms upon rechallenge.

The DLST was performed using the initial 5‐ASA formulation suspected to have caused intolerance and performed by an external laboratory (LSI Medience Corporation, Tokyo, Japan). Lymphocyte proliferation was expressed as a stimulation index (SI). SI values < 180% were defined as negative, 180–199% as borderline, and ≥ 200% as positive.

The diagnosis of 5‐ASA intolerance was based on routine clinical documentation by treating gastroenterologists, with symptoms evaluated and discussed within the IBD care team. Electronic medical records were systematically reviewed, and the predefined diagnostic criteria were applied to classify intolerance.

To attribute symptoms to 5‐ASA intolerance, infectious and other alternative causes were excluded by stool cultures, *C. difficile* toxin testing, and other appropriate laboratory and imaging evaluations. When necessary, patients were additionally reviewed by relevant specialists.

Rechallenge to assess reproducibility was performed using the initial or alternative 5‐ASA formulations, including sulfasalazine and topical preparations. It was attempted only in patients with mild to moderate intolerance without organ toxicities such as pneumonitis, pancreatitis, or severe skin reactions after informed consent, either by switching formulations or by rechallenging the initial drug at the minimum dose under close monitoring.

Tolerance on rechallenge was defined as the ability to continue 5‐ASA treatment for at least 3 months at or above the standard maintenance dose without recurrence of intolerance symptoms.

The observation period was defined as the time from initiation of oral 5‐ASA therapy to the last follow‐up visit. Patients who discontinued 5‐ASA were regarded as having experienced the event, whereas those who continued were censored at their last follow‐up.

### 2.5 Statistical Analysis

Patient background data were retrospectively extracted from electronic medical records at the time of initiation of 5‐ASA therapy. Baseline variables included age, sex, body mass index, disease duration and extent, clinical and endoscopic activity indices, laboratory data, smoking status, comorbidities, concomitant medications, and 5‐ASA treatment details.

C‐reactive protein (CRP) and erythrocyte sedimentation rate (ESR) were not available for all patients at baseline; analyses were performed using complete cases without imputation.

Cutoff values were determined based on receiver operating characteristic curve (ROC) analysis (age, CRP) in the intolerance analysis or established clinical severity criteria (ESR) [21]. For CRP, the conventional cutoff of 3.0 mg/dL led to separation with no intolerance cases in the higher group; therefore, the ROC‐derived cutoff of 0.3 mg/dL was used.

Variables included in the multivariate models were selected based on clinical relevance and results of the univariate analyses. The number of covariates included in each multivariable model was limited according to the number of events.

GraphPad Prism 10 software package (GraphPad Software Inc., San Diego, CA, USA) was used to draw the graph of the continuation rate. All statistical analyses were performed using EZR (Saitama Medical Center, Jichi Medical University, Saitama, Japan), which is a graphical user interface for R (R Foundation for Statistical Computing, Vienna, Austria). 5‐ASA continuation was analyzed using the Kaplan–Meier method with comparisons by the log‐rank test, and discontinuation risk was assessed using univariate and multivariable Cox proportional hazards models. For 5‐ASA intolerance, univariate and multivariable logistic regression analyses were performed. As a sensitivity analysis, Firth penalized logistic regression was performed using the logistf package in R.

Significant differences between outcomes were indicated by a *p* value less than 0.05.

## 3 Results

### 3.1 Patient Registration

**FIGURE 1:**
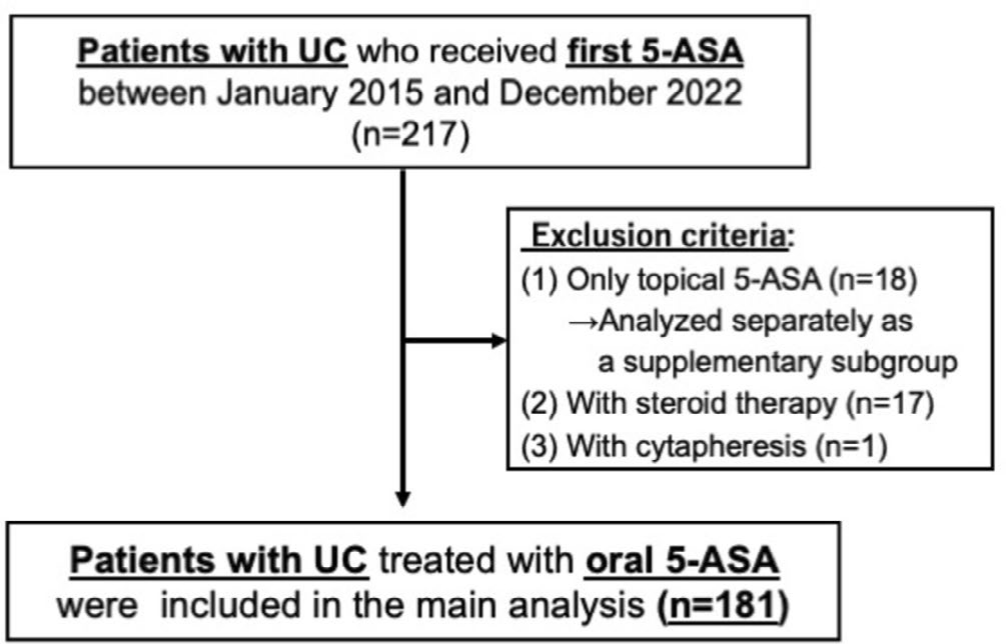
Patient selection. 5‐ASA, 5‐aminosalicylic acid; UC, ulcerative colitis.

Between January 2015 and December 2022, 217 patients with UC received their first 5‐ASA treatment at our hospital. Of these, the following patients were excluded: 18 patients who were treated with only topical 5‐ASA, 17 patients who received their first 5‐ASA concomitantly with steroids (budesonide enema or oral prednisolone), and 1 patient who received 5‐ASA concomitantly with cytapheresis. The remaining 181 patients with UC were enrolled (Figure 1).

### 3.2 Patients' Background

The clinical characteristics of the patients are summarized in Table 1. The median follow‐up duration was 58 months. Eighty‐five patients (47.0%) were followed up for ≥ 5 years. At the start of the 5‐ASA, a PPI or PCAB was concomitantly used in 10 (5.5%) patients. The median ages of the PPI/PCAB non‐used and PPI/PCAB groups were 37 (12–74) years and 58.5 (25–79) years, respectively. In each group, the median partial Mayo score was 4 (0–8) and 4.5 (0–6), and the median serum CRP level was 0.06 (0.01–4.39) mg/dL and 0.45 (0.03–4.5) mg/dL, respectively. In the PPI/PCAB group, eight and two patients were prescribed time‐dependent and multi‐matrix system (MMX) formulations of 5‐ASA. PPIs were concomitantly used in eight patients, and PCAB was used in two patients. Detailed clinical information for all PPI/PCAB patients is provided in Table S1.

**TABLE 1:** Patient characteristics at the time of 5‐ASA initiation.

Characteristics	Non‐PPI/PCAB group, *n* = 171	PPI/PCAB group, *n* = 10 (5.5%)	*p*
Observation period, months, median (range)	58 (0–119)		
Age (years), median (range, SD)	37 (12–74, 14.9)	58.5 (25–79, 17.5)	< 0.01
Male/female, *n*	92/79	6/4	0.76
Current smoking, *n* (%)	27 (15.8%)	0 (0%)	0.36
Body mass index, median (range)	23.2 (17.8–28.9)	22.0 (15.4–33.3)	0.51
Proctitis/left side/pancolitis, *n*	60/43/67	6/0/4	
Partial Mayo score, median (range)	4 (0–8)	4.5 (0–6)	0.39
Endoscopic Mayo score 1/2/3, *n*	22/143/6	0/9/1	0.13
CAI (Rachmilewitz), median (range)	4 (0–18)	4 (1–7)	0.63
CRP (mg/dL), median (range)	0.06 (0.01–4.39)	0.45 (0.03–4.5)	< 0.01
ESR (mm/h), median (range)	12 (1–93)	20.0 (4–92)	0.16
Oral formulations of 5‐ASA Time‐dependent/pH‐dependent/MMX, *n* (%)	113/16/42	8/0/2	
Oral 5‐ASA dose, mg, median (range)	4000 (2000–4800)	4000 (2000–4800)	0.57
Oral + topical 5‐ASA, *n* (%)	35 (19.3%)	3 (30%)	0.44

*Note:* Continuous variables are compared using the Mann–Whitney *U* test. Categorical variables are compared using the Fisher's exact test.

Abbreviations: 5‐ASA, 5‐aminosalicylic acid; CAI, clinical activity index; CRP, C‐reactive protein; ESR, erythrocyte sedimentation rate; MMX, multi‐matrix system; PCAB, potassium competitive acid blocker; PPI, proton pump inhibitor; SD, standard deviation.

### 3.3 5‐ASA Discontinuation Analysis

Among the 181 patients analyzed, 101 (55.8%) discontinued their initial oral 5‐ASA therapy for the following reasons: 29 (28.7%) due to intolerance; 12 (11.9%) due to primary nonresponse; 36 (35.6%) secondary loss of response; 5 (5.0%) endoscopic nonresponse; and 19 (18.8%) suspected intolerance that did not meet the criteria for intolerance (Table 2).

**TABLE 2:** Reasons for discontinuation of initial oral 5‐ASA therapy (*n* = 101).

Reason for discontinuation	*n (%)*	Notes
Intolerance	29 (28.7)	Met predefined criteria
Primary nonresponse	12 (11.9)	Lack of initial efficacy
Secondary loss of response	36 (35.6)	Relapse after initial response
Endoscopic nonresponse	5 (5.0)	No endoscopic improvement
Suspected intolerance (not meeting intolerance criteria)	19 (18.8)	3 not re‐challenged, 16 tolerated on re‐administration

Abbreviation: 5‐ASA, 5‐aminosalicylic acid.

**FIGURE 2:**
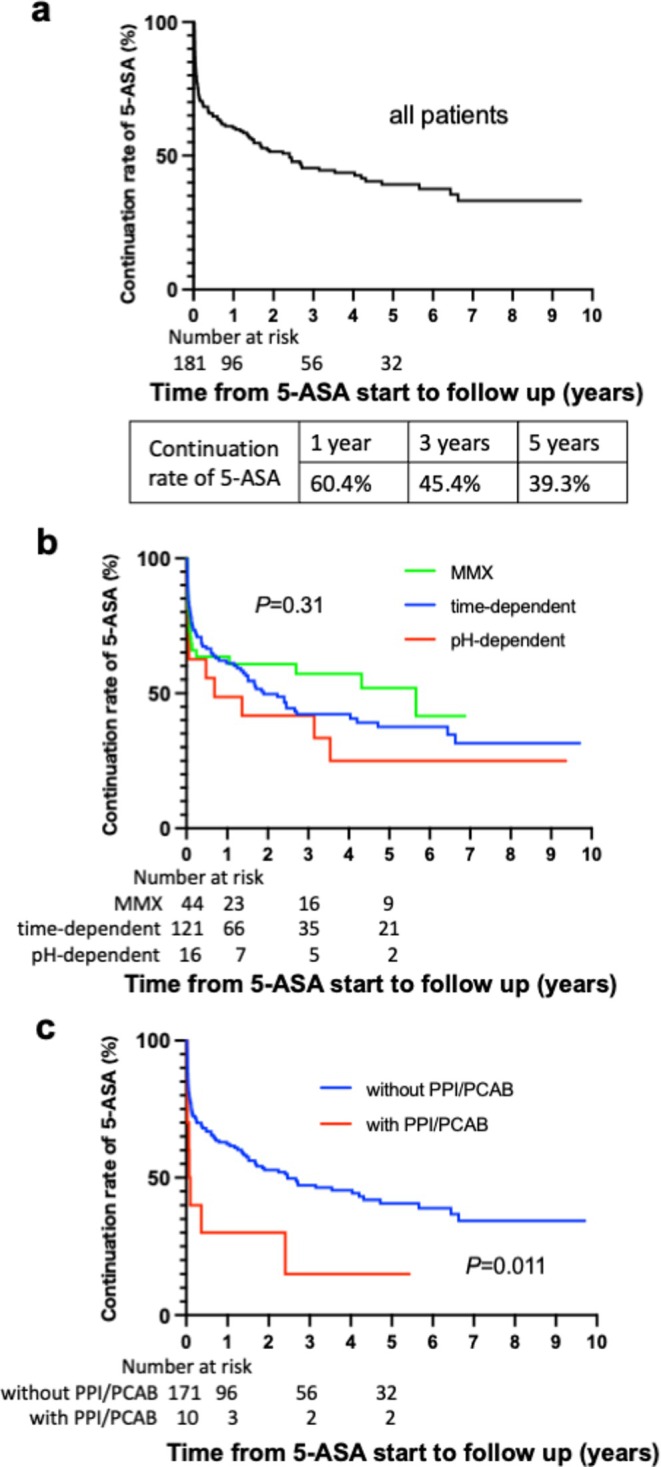
Kaplan–Meier curve for continuation rate of the first oral 5‐ASA. (a) Continuation rate of 5‐ASA in all patients. (b) Comparison of continuation rates among MMX, time‐dependent, and pH‐dependent formulations. (c) Comparison of continuation rates between patients with and without concomitant PPI/PCAB use. *p* value was determined using a log‐rank test. 5‐ASA, 5‐aminosalicylic acid; MMX, multimatrix system; PCAB, potassium‐competitive acid blocker; PPI, proton pump inhibitor.

In all patient groups, the cumulative probabilities of maintaining the first oral 5‐ASA treatment at 1, 3, and 5 years were 60.4%, 45.4%, and 39.3%, respectively (Figure 2a). Kaplan–Meier analysis showed no significant differences in the continuation rates of the time‐dependent, pH‐dependent, and MMX formulations (*p* = 0.31) (Figure 2b). The 5‐ASA continuation rate was significantly lower in the PPI/PCAB group than in the non‐PPI/PCAB group (*p* = 0.011) (Figure 2c). In univariate analysis, PPI/PCAB use was the only variable significantly associated with first oral 5‐ASA discontinuation (hazard ratio [HR] 2.47, 95% confidence interval [CI] 1.19–5.10, *p* = 0.014) (Table 3). In multivariate analysis, PPI/PCAB use remained independently associated with 5‐ASA discontinuation (HR 2.46, 95% CI 1.05–5.65, *p* = 0.034). No significant associations were observed between discontinuation and disease activity, disease extent, or 5‐ASA formulation. A total of 101 discontinuation events occurred, yielding an events‐per‐variable ratio of approximately 20 in the multivariable Cox model.

**TABLE 3:** Univariate and multivariate analysis of 5‐ASA discontinuation.

Variables	Univariate analysis			Multivariate analysis		
	HR	95% CI	*p*	HR	95% CI	*p*
Age ≥ 40 years	0.94	0.64–1.39	0.76	0.82	0.54–1.25	0.36
Male	1.27	0.85–1.88	0.24	0.83	0.55–1.25	0.38
Current smoking	1.18	0.68–2.05	0.55			
Obesity (BMI ≥ 25)	1.40	0.85–2.28	0.18			
PPI/PCAB use	2.47	1.19–5.10	0.014	2.46	1.06–5.65	0.034
Pancolitis	1.04	0.70–1.56	0.84	0.94	0.59–1.51	0.81
Partial Mayo score ≥ 3	1.35	0.86–2.13	0.19			
Endoscopic Mayo score 3	1.18	0.42–3.20	0.75			
CAI (Rachmilewitz) ≥ 5	1.01	0.67–1.52	0.98			
CRP ≥ 0.3 mg/dL	1.08	0.66–1.76	0.73	0.99	0.58–1.72	0.99
ESR ≥ 30 mm/h	0.98	0.58–1.66	0.93			
MMX	0.77	0.47–1.26	0.29			
Combined use of topical 5‐ASA	0.91	0.57–1.45	0.70			

*Note:* Hazard ratio (HR) and 95% confidence interval (CI) were calculated using the Cox proportional hazards model.

Abbreviations: 5‐ASA, 5‐aminosalicylic acid; BMI, body mass index; CAI, clinical activity index; CRP, C‐reactive protein; ESR, erythrocyte sedimentation rate; MMX, multi‐matrix system; PCAB, potassium competitive acid blocker; PPI, proton pump inhibitor.

As a supplementary analysis excluding intolerance‐related discontinuations, no significant difference in non–intolerance‐related 5‐ASA discontinuation was observed between the PPI/PCAB and non‐PPI/PCAB groups (*p* = 0.47) (Figure S1).

### 3.4 5‐ASA Intolerance Analysis

In all patients, the 5‐ASA intolerance rate was 16.0% (29/181) (Table 4). Of the 29 patients with 5‐ASA intolerance, 16 fulfilled the temporal criteria with characteristic symptoms resolving after drug withdrawal. Intolerance was confirmed by a positive DLST in a patient, and by rechallenge in 12 patients. The diagnostic process for confirming 5‐ASA intolerance is summarized in Figure S2. In univariate analysis, patients' age ≥ 40 years was significantly associated with reduced odds of 5‐ASA intolerance (odds ratio [OR] 0.41, 95% CI 0.17–0.94, *p* = 0.037), whereas concomitant PPI/PCAB use was associated with increased odds (OR 6.12, 95% CI 1.65–22.8, *p* < 0.01) (Table 4). In the multivariate model, both factors remained significant: age ≥ 40 years was associated with reduced odds of intolerance (OR 0.21, 95% CI 0.15–0.47, *p* < 0.01), while PPI/PCAB use was associated with increased odds (OR 9.65, 95% CI 1.92–48.5, *p* < 0.01). No significant associations were observed between 5‐ASA intolerance and disease activity, disease extent, or 5‐ASA formulation. In the multivariable Firth's penalized logistic regression model, age ≥ 40 years was associated with a significantly higher odds of 5‐ASA intolerance (OR 0.24, 95% CI 0.08–0.61, *p* < 0.01), whereas concomitant PPI/PCAB use was associated with higher odds of 5‐ASA intolerance (OR 8.81, 95% CI 1.90–42.8, *p* < 0.01) (Table S2). A total of 29 intolerance events occurred, yielding an events‐per‐variable ratio of approximately 9.7 in the multivariable logistic regression analysis.

**TABLE 4:** Univariate and multivariate analysis of 5‐ASA intolerance (29/181 [16.0%]).

Variables	Univariate analysis			Multivariate analysis		
	OR	95% CI	*p*	OR	95% CI	*p*
Age ≥ 40 years	0.41	0.17–0.94	0.037	0.21	0.15–0.47	< 0.01
Male	0.89	0.40–1.97	0.78			
Current smoking	0.89	0.29–2.82	0.85			
Allergic disease	0.55	0.20–1.55	0.26			
Drug allergy history	0.71	0.15–3.30	0.66			
Obesity (BMI ≥ 25)	0.89	0.28–2.80	0.84			
PPI/PCAB use	6.12	1.65–22.8	< 0.01	9.65	1.92–48.5	< 0.01
H1 blocker use	1.48	0.38–5.67	0.57			
Pancolitis	0.79	0.34–1.80	0.57			
Partial Mayo score ≥ 3	2.30	0.82–6.41	0.11			
Endoscopic Mayo score 3	0.87	0.10–7.49	0.90			
CAI (Rachmilewitz) ≥ 5	1.16	0.52–2.58	0.73			
CRP ≥ 0.3 mg/dL	1.32	0.51–3.41	0.57	0.99	0.35–2.79	0.99
ESR ≥ 30 mm/h	0.71	0.27–2.22	0.55			
MMX	0.99	0.39–2.50	0.98			
Concomitant use of topical 5‐ASA	0.75	0.27–2.12	0.59			
Oral 5‐ASA dose ≥ 4000 mg	1.83	0.65–5.12	0.24			

*Note:* Odds ratio (OR) and 95% confidence interval (CI) were calculated using logistic regression analysis.

Abbreviations: 5‐ASA, 5‐aminosalicylic acid; BMI, body mass index; CAI, clinical activity index; CRP, C‐reactive protein; ESR, erythrocyte sedimentation rate; MMX, multi‐matrix system; PCAB, potassium competitive acid blocker; PPI, proton pump inhibitor.

### 3.5 Supplementary Analysis

Among 18 patients treated exclusively with topical 5‐ASA (enema, *n* = 3; suppository, *n* = 15), one received concomitant PPI/PCAB, and one developed intolerance (suppository user without concomitant PPI/PCAB) (Table S3).

## 4 Discussion

In this study, we investigated factors associated with discontinuation and intolerance of oral 5‐ASA in patients with UC. We found that PPI/PCAB use was associated with both discontinuation and intolerance of oral 5‐ASA. The present study is the first to analyze the effect of PPI/PCAB on 5‐ASA intolerance in patients with UC.

Previous studies have suggested associations between PPI use and multiple aspects of UC. First, PPI use has been linked to an increased likelihood of incident UC [22]. In terms of treatment efficacy, concomitant PPI use has been associated with lower effectiveness of infliximab and vedolizumab in patients with IBD [23, 24]. At the level of clinical outcomes, PPI use was associated with an increased likelihood of initiating glucocorticoid treatment in patients with UC [19]. Moreover, PPI use has been associated with an increased likelihood of hospitalization or surgery in patients with UC [9]. However, a causal relationship has not been established.

The mechanisms underlying the association between PPI/PCAB use and 5‐ASA intolerance remain unclear, but several possibilities have been suggested. First, pharmacokinetic interactions may be involved. Van Camp et al. demonstrated that PPI pretreatment accelerated 5‐ASA absorption and increased systemic exposure [25]. They suggested that PPI‐induced faster 5‐ASA release and increased systemic absorption may reduce colonic delivery and increase adverse event risk. Moreover, for pH‐dependent and MMX formulations, PPI/PCAB‐induced increases in intragastric and intestinal pH may accelerate proximal dissolution, potentially contributing to altered exposure and intolerance. Although we found no significant differences in discontinuation by 5‐ASA formulation, Shimodaira et al. reported that PPIs or H_2_‐receptor antagonists coadministration increased relapse risk in patients using pH‐dependent 5‐ASA [26]. This discrepancy may be explained by differences in study design and formulation distribution, as none of our PPI/PCAB users received a pH‐dependent formulation.

Second, alterations in the gut microbiota could be relevant. Large cohort studies have shown that PPI administration is associated with reduced α‐diversity [27, 28] and an increased abundance of *Streptococcus* species [12, 29], while PCAB use may induce similar or greater shifts [30]. In parallel, Mizuno et al. reported that patients with 5‐ASA intolerance displayed increased abundance of *Streptococcus*, *Faecalibacterium*, and *Clostridium* [7]. Dysbiosis has also been implicated in UC pathogenesis, with reduced microbial diversity and pathobiont expansion contributing to immune dysregulation and barrier dysfunction [31, 32, 33]. These alterations may promote aberrant mucosal immune activation and increase susceptibility to injury, thereby linking PPI/PCAB‐induced microbial shifts to 5‐ASA intolerance.

In addition to drug‐related mechanisms, younger age was independently associated with 5‐ASA intolerance in our cohort, consistent with previous reports [5]. Greater immunoreactivity in younger individuals, including higher thymic output and limited prior drug exposure, may increase susceptibility to hypersensitivity reactions and partly explain this association [34].

Lichtenstein et al. reported that concomitant PPI use did not appear to affect the efficacy of pH‐dependent 5‐ASA in patients with UC [20]. However, their study focused only on treatment efficacy and did not examine discontinuation or intolerance. In addition, their analysis was limited to pH‐dependent formulations, whereas our study also included time‐dependent and MMX formulations. Furthermore, their cohort conducted in the United States between 2007 and 2011 differed substantially in terms of the study period and background characteristics. These differences may help explain the discrepant findings between their study and the present study.

In our single‐center cohort, the overall 5‐ASA discontinuation rate appeared relatively high. This may reflect differences in patient characteristics, definitions of discontinuation, and real‐world physician decision‐making in our institution. The proportion of confirmed 5‐ASA intolerance was 16.0%, which is comparable to Japanese real‐world data reporting rates up to 16.2% between 2014 and 2016 [5].

A major strength of this study is the strict inclusion of 5‐ASA–naïve patients without concurrent steroid or other UC therapies, minimizing masking of intolerance symptoms. In addition, intolerance was rigorously defined, with tolerance on rechallenge excluding the diagnosis.

This study has some limitations.

1. This was a single‐center, retrospective, observational study. Decisions regarding 5‐ASA discontinuation may have varied among physicians, and reasons for discontinuation were not uniformly documented, potentially reflecting physician decision‐making rather than drug efficacy alone.
2. The number of patients in the PPI/PCAB group was very small (*n* = 10), resulting in limited statistical power and wide CIs, and the estimates should therefore be interpreted with caution. Moreover, recent PPI/PCAB use among non‐users may have influenced microbiota‐related outcomes or intolerance risk [29].
3. Residual confounding cannot be excluded, particularly regarding comorbidities or the clinical indications that led to PPI/PCAB use.
4. Because this study was conducted at a single institution in Japan, the findings may not be directly generalizable to other populations or healthcare settings.
5. Because many patients were prescribed PPIs/PCABs at outside institutions, the exact timing of initiation or discontinuation during follow‐up could not be reliably determined. As exposure was defined only at baseline, misclassification of time‐varying PPI/PCAB use cannot be fully excluded.

Protopathic bias is unlikely, as PPI/PCAB use was defined at the time of initial 5‐ASA initiation in treatment‐naïve UC and therefore was not introduced in response to intolerance or disease exacerbation. Nevertheless, owing to the above limitations, our findings should be interpreted in the context of the study design.

In conclusion, PPI/PCAB use was associated with oral 5‐ASA discontinuation and intolerance in patients with UC. Given the retrospective design and small number of PPI/PCAB users, these findings should be interpreted as associations, and larger multicenter studies are needed for validation.

## Funding

The authors have nothing to report.

## Ethics Statement

We conducted this study in compliance with the principles of the Declaration of Helsinki. This study was approved by the Human Research Ethics Committee of the Sapporo Higashi Tokushukai Hospital (IRB No. TGE 01386‐012).

## Consent

Informed consent was waived by the ethics committee owing to the retrospective nature of the study and use of anonymized data. The study protocol was posted on the study websites. Patients opted out of the study if they did not wish to provide consent.

## Conflicts of Interest

Shinsuke Otagiri, Takahiro Ito, and Atsuo Maemoto received a research grant from AbbVie GK, Janssen Pharmaceutical K.K., Ely Lilly Japan K.K., and Pfizer R&D Japan G.K. Takahiro Ito received lecture fees from Janssen Pharmaceutical K.K. The other authors declare no conflicts of interest.

## Supporting information


**Table S1:** Clinical characteristics and treatment course of patients using PPI/PCAB.
**Table S2:** Firth penalized logistic regression analysis for 5‐ASA intolerance.
**Table S3:** Characteristics of patients treated with topical 5‐ASA only.

**Figure S1:** Kaplan–Meier curve of 5‐ASA continuation excluding intolerance‐related discontinuations. *p* value was determined using a log‐rank test. 5‐ASA, 5‐aminosalicylic acid; PCAB, potassium‐competitive acid blocker; PPI, proton pump inhibitor.

**Figure S2:** Diagnostic flow of 5‐ASA intolerance. ASA, 5‐aminosalicylic acid; DLST, drug lymphocyte stimulation test.

## Data Availability

The data that support the findings of this study are available from the corresponding author upon reasonable request.

## References

[1] H. Chu , A. Khosravi , I. P. Kusumawardhani , et al., “Gene‐Microbiota Interactions Contribute to the Pathogenesis of Inflammatory Bowel Disease,” Science 352, no. 6289 (2016): 1116–1120, 10.1126/science.aad9948. 27230380 PMC4996125

[2] B. E. Sands , W. J. Sandborn , R. Panaccione , et al., “Ustekinumab as Induction and Maintenance Therapy for Ulcerative Colitis,” New England Journal of Medicine 381, no. 13 (2019): 1201–1214, 10.1056/NEJMoa1900750. 31553833

[3] W. J. Sandborn , C. Su , B. E. Sands , et al., “Tofacitinib as Induction and Maintenance Therapy for Ulcerative Colitis,” New England Journal of Medicine 376, no. 18 (2017): 1723–1736, 10.1056/NEJMoa1606910. 28467869

[4] R. Ungaro , S. Mehandru , P. B. Allen , L. Peyrin‐Biroulet , and J. F. Colombel , “Ulcerative Colitis,” Lancet 389, no. 10080 (2017): 1756–1770, 10.1016/S0140-6736(16)32126-2. 27914657 PMC6487890

[5] S. Hiraoka , A. Fujiwara , T. Toyokawa , et al., “Multicenter Survey on Mesalamine Intolerance in Patients With Ulcerative Colitis,” Journal of Gastroenterology and Hepatology 36, no. 1 (2021): 137–143, 10.1111/jgh.15138. 32525567

[6] Y. Mikami , J. Tsunoda , S. Suzuki , I. Mizushima , H. Kiyohara , and T. Kanai , “Significance of 5‐Aminosalicylic Acid Intolerance in the Clinical Management of Ulcerative Colitis,” Digestion 104, no. 1 (2023): 58–65, 10.1159/000527452. 36366816 PMC9843541

[7] S. Mizuno , K. Ono , Y. Mikami , et al., “5‐Aminosalicylic Acid Intolerance Is Associated With a Risk of Adverse Clinical Outcomes and Dysbiosis in Patients With Ulcerative Colitis,” Intestinal Research 18, no. 1 (2020): 69–78, 10.5217/ir.2019.00084. 32013315 PMC7000647

[8] H. Matsumoto , M. Sasahira , T. T. Go , et al., “Characteristics of Mucosa‐Associated Microbiota in Ulcerative Colitis Patients With 5‐Aminosalicylic Acid Intolerance,” Biomedicine 12, no. 9 (2024): 2125, 10.3390/biomedicines12092125. PMC11428711 39335641

[9] T. Choden , H. Zhang , and A. Sakuraba , “Influence of Proton Pump Inhibitor Use on Clinical Outcomes of Patients With Inflammatory Bowel Disease,” Annals of Medicine 55, no. 1 (2023): 2198775, 10.1080/07853890.2023.2198775. 37070427 PMC10124315

[10] D. M. Simadibrata , A. F. Syam , and Y. Y. Lee , “A Comparison of Efficacy and Safety of Potassium‐Competitive Acid Blocker and Proton Pump Inhibitor in Gastric Acid‐Related Diseases: A Systematic Review and Meta‐Analysis,” Journal of Gastroenterology and Hepatology 37, no. 12 (2022): 2217–2228, 10.1111/jgh.16017. 36181401 PMC10092067

[11] H. Kaneko , H. Sato , Y. Suzuki , et al., “A Novel Characteristic Gastric Mucus Named “Web‐Like Mucus” Potentially Induced by Vonoprazan,” Journal of Clinical Medicine 13, no. 14 (2024): 4070, 10.3390/jcm13144070. 39064109 PMC11277586

[12] T. Takagi , Y. Naito , R. Inoue , et al., “The Influence of Long‐Term Use of Proton Pump Inhibitors on the Gut Microbiota: An Age‐Sex‐Matched Case‐Control Study,” Journal of Clinical Biochemistry and Nutrition 62, no. 1 (2018): 100–105, 10.3164/jcbn.17-78. 29371761 PMC5773837

[13] M. L. Ouyang , S. P. Zou , Q. Cheng , X. Shi , Y. Z. Zhao , and M. H. Sun , “Effect of Potassium‐Competitive Acid Blockers on Human Gut Microbiota: A Systematic Review and Meta‐Analysis,” Frontiers in Pharmacology 14 (2023): 1269125, 10.3389/fphar.2023.1269125. 38192408 PMC10773775

[14] T. Dong and J. Pisegna , “Passing the “Acid Test”: Do Proton Pump Inhibitors Affect the Composition of the Microbiome?,” Digestive Diseases and Sciences 63, no. 11 (2018): 2817–2819, 10.1007/s10620-018-5273-3. 30218426 PMC6408263

[15] S. M. Lee , N. Kim , R. H. Nam , et al., “Gut Microbiota and Butyrate Level Changes Associated With the Long‐Term Administration of Proton Pump Inhibitors to Old Rats,” Scientific Reports 9, no. 1 (2019): 6626, 10.1038/s41598-019-43112-x. 31036935 PMC6488615

[16] M. Nighot , P. L. Liao , N. Morris , et al., “Long‐Term Use of Proton Pump Inhibitors Disrupts Intestinal Tight Junction Barrier and Exaggerates Experimental Colitis,” Journal of Crohn's & Colitis 17, no. 4 (2023): 565–579, 10.1093/ecco-jcc/jjac168. PMC10115233 36322638

[17] Y. Furusawa , Y. Obata , S. Fukuda , et al., “Commensal Microbe‐Derived Butyrate Induces the Differentiation of Colonic Regulatory T Cells,” Nature 504, no. 7480 (2013): 446–450, 10.1038/nature12721. 24226770

[18] B. Xia , M. Yang , L. H. Nguyen , et al., “Regular Use of Proton Pump Inhibitor and the Risk of Inflammatory Bowel Disease: Pooled Analysis of 3 Prospective Cohorts,” Gastroenterology 161, no. 6 (2021): 1842–1852.e10, 10.1053/j.gastro.2021.08.005. 34389338

[19] R. Fossmark , S. S. Lirhus , and M. L. Høivik , “The Impact of Proton Pump Inhibitors on the Course of Ulcerative Colitis: A Cohort Study of Over 10,000 Newly Diagnosed Patients in Norway,” Scandinavian Journal of Gastroenterology 59, no. 1 (2024): 46–51, 10.1080/00365521.2023.2255710. 37681998

[20] G. Lichtenstein , R. Marchioni , W. Blonski , P. Mudireddy , and A. Buchner , “Efficacy of Oral Mesalamine Monotherapy (5‐ASA) Versus Combination Oral Mesalamine Plus Proton Pump Inhibitor (5‐ASA/PPI) Therapy in the Treatment of Ulcerative Colitis (UC),” American Journal of Gastroenterology 107 (2012): S630–S631, 10.14309/00000434-201210001-01565.

[21] H. Nakase , M. Uchino , S. Shinzaki , et al., “Evidence‐Based Clinical Practice Guidelines for Inflammatory Bowel Disease 2020,” Journal of Gastroenterology 56, no. 6 (2021): 489–526, 10.1007/s00535-021-01784-1. 33885977 PMC8137635

[22] Y. Liang , Z. Meng , X. L. Ding , and M. Jiang , “Effects of Proton Pump Inhibitors on Inflammatory Bowel Disease: An Updated Review,” World Journal of Gastroenterology 30, no. 21 (2024): 2751–2762, 10.3748/wjg.v30.i21.2751. 38899331 PMC11185295

[23] T. X. Lu , M. Dapas , E. Lin , T. Peters , and A. Sakuraba , “The Influence of Proton Pump Inhibitor Therapy on the Outcome of Infliximab Therapy in Inflammatory Bowel Disease: A Patient‐Level Meta‐Analysis of Randomised Controlled Studies,” Gut 70, no. 11 (2021): 2076–2084, 10.1136/gutjnl-2020-321609. 33334900

[24] K. Szemes , N. Farkas , Z. Sipos , et al., “Co‐Administration of Proton Pump Inhibitors May Negatively Affect the Outcome in Inflammatory Bowel Disease Treated With Vedolizumab,” Biomedicine 12, no. 1 (2024): 158, 10.3390/biomedicines12010158. PMC10813460 38255263

[25] A. Van Camp , T. Vanuytsel , J. Brouwers , and P. Augustijns , “The Effect of Esomeprazole on the Upper GI Tract Release and Systemic Absorption of Mesalazine From Colon Targeted Formulations,” International Journal of Pharmaceutics 619 (2022): 121701, 10.1016/j.ijpharm.2022.121701. 35339635

[26] Y. Shimodaira , K. Onochi , K. Watanabe , et al., “Effect of Acid‐Reducing Agents on Clinical Relapse in Ulcerative Colitis With pH‐Dependent‐Released 5‐Aminosalicylic Acid: A Multicenter Retrospective Study in Japan,” Intestinal Research 19, no. 2 (2021): 225–231, 10.5217/ir.2020.00023. 32806877 PMC8100376

[27] F. Imhann , M. J. Bonder , A. Vich Vila , et al., “Proton Pump Inhibitors Affect the Gut Microbiome,” Gut 65, no. 5 (2016): 740–748, 10.1136/gutjnl-2015-310376. 26657899 PMC4853569

[28] M. A. Jackson , J. K. Goodrich , M. E. Maxan , et al., “Proton Pump Inhibitors Alter the Composition of the Gut Microbiota,” Gut 65, no. 5 (2016): 749–756, 10.1136/gutjnl-2015-310861. 26719299 PMC4853574

[29] D. E. Freedberg , N. C. Toussaint , S. P. Chen , et al., “Proton Pump Inhibitors Alter Specific Taxa in the Human Gastrointestinal Microbiome: A Crossover Trial,” Gastroenterology 149, no. 4 (2015): 883–885.e9, 10.1053/j.gastro.2015.06.043. 26164495 PMC4584196

[30] T. Otsuka , M. Sugimoto , R. Inoue , et al., “Influence of Potassium‐Competitive Acid Blocker on the Gut Microbiome of *Helicobacter pylori*‐Negative Healthy Individuals,” Gut 66, no. 9 (2017): 1723–1725, 10.1136/gutjnl-2016-313312. 27965281

[31] A. D. Kostic , R. J. Xavier , and D. Gevers , “The Microbiome in Inflammatory Bowel Disease: Current Status and the Future Ahead,” Gastroenterology 146, no. 6 (2014): 1489–1499, 10.1053/j.gastro.2014.02.009. 24560869 PMC4034132

[32] J. Ni , G. D. Wu , L. Albenberg , and V. T. Tomov , “Gut Microbiota and IBD: Causation or Correlation?,” Nature Reviews Gastroenterology & Hepatology 14, no. 10 (2017): 573–584, 10.1038/nrgastro.2017.88. 28743984 PMC5880536

[33] T. Zuo and S. C. Ng , “The Gut Microbiota in the Pathogenesis and Therapeutics of Inflammatory Bowel Disease,” Frontiers in Microbiology 9 (2018): 2247, 10.3389/fmicb.2018.02247. 30319571 PMC6167487

[34] J. M. Murray , G. R. Kaufmann , P. D. Hodgkin , et al., “Naive T Cells Are Maintained by Thymic Output in Early Ages but by Proliferation Without Phenotypic Change After Age Twenty,” Immunology and Cell Biology 81, no. 6 (2003): 487–495, 10.1046/j.1440-1711.2003.01191.x. 14636246

