# Supplementary material for: Association of Proton Pump Inhibitor and Potassium‐Competitive Acid Blocker Use With Discontinuation and Intolerance of Oral 5‐Aminosalicylic Acid in Patients With Ulcerative Colitis
Source: JGH Open. 2026 Jan 31;10(2):e70350. doi: 10.1002/jgh3.70350 (PMC12860887; doi:10.1002/jgh3.70350)
Supplement: Supplementary file 1 — Table S1: Clinical characteristics and treatment course of patients using PPI/PCAB. Table S2: Firth penalized logistic regression analysis for 5‐ASA intolerance. Table S3: Characteristics of patients treated with topical 5‐ASA only. [file JGH3-10-e70350-s002.docx]

Supplementary Table 1. Clinical characteristics and treatment course of patients using PPI/PCAB

|  | **Case 1** | **Case 2** | **Case 3** | **Case 4** | **Case 5** | **Case 6** | **Case 7** | **Case 8** | **Case 9** | **Case 10** |
| --- | --- | --- | --- | --- | --- | --- | --- | --- | --- | --- |
| **Age** | 56 | 87 | 63 | 56 | 48 | 63 | 78 | 32 | 61 | 25 |
| **Sex** | Female | Female | Male | Male | Male | Male | Male | Female | Male | Male |
| **Indication for PPI/PCAB** | Unknown | With aspirin | With aspirin | Unknown | IBS | unknown | unknown | FD | unknown | GERD |
| **PPI or PCAB** | Omeprazole | Esomeprazole | Lansoprazole | Rabeprazole | Rabeprazole | Rabeprazole | Esomeprazole | Esomeprazole | Vonoprazan | Vonoprazan |
| **Exposure length of PPI/PCAB** | Unknown | Unknown | Unknown | Unknown | Unknown | Unknown | Unknown | 3 days | Unknown | Unknow |
| **Disease extent** | Pancolitis | Pancolitis | Pancolitis | Proctitis | Proctitis | Proctitis | Pancolitis | Pancolitis | Pancolitis | Proctitis |
| **Upper GI involvement** | No | No | No | Not available | No | No | No | Not available | No | Not available |
| **Partial Mayo score at baseline** | 6 | 5 | 4 | 0 | 3 | 2 | 5 | 6 | 6 | 3 |
| **5-ASA formulation**  **(dosage)** | Time-dependent  (4000 mg) | Time-dependent  (4000 mg) | Time-dependent  (4000 mg) | Time-dependent  (4000 mg) | Time-dependent  (4000 mg) | Time-dependent  (2000 mg) | MMX  (4800mg) | Time-dependent  (4000 mg) | MMX  (4800 mg) | Time-dependent  (4000 mg) |
| **5-ASA discontinuation status (timing)** | Yes  (131 days) | Yes  (878 days) | No | Yes  (10 days) | Yes,  (35 days | Yes  (8 days) | No | Yes  (23 days) | Yes  (28 days) | Yes  (8 days) |
| **Reason for discontinuation** | Loss of response | Endoscopic nonresponse |  | Intolerance suspected | Intolerance suspected | Intolerance suspected |  | Intolerance suspected | Intolerance suspected | Intolerance suspected |
| **5-ASA intolerance** | No | No | No | Yes | Yes | Yes | No | Yes | No | Yes |
| **Symptoms** |  |  |  | Fever, diarrhea | Abdominal pain, diarrhea | Abdominal pain, diarrhea |  | Pancreatitis |  | Fever, diarrhea |
| **Diagnosis of intolerance** |  |  |  | Temporal criteria | Rechallenge | Temporal criteria |  | Temporal criteria |  | Rechallenge |
| **PPI/PCAB continued at event** |  |  |  | Not available | Continue | Not available |  | Continue |  | Continue |

PPI, proton pump inhibitor; PCAB, potassium competitive acid blocker; IBS, irritable bowel syndrome; FD, functional dyspepsia; GERD, gastroesophageal reflux disease; GI, gastrointestinal; 5-ASA, 5-aminosalicylic acid; MMX, multi-matrix system.

Supplementary Table 2. Firth penalized logistic regression analysis for 5-ASA intolerance

| **Variables** | **Coefficient (β)** | **OR (95% CI)** | ***P*** |
| --- | --- | --- | --- |
| Age≥40 years | -1.42 | 0.24 (0.08-0.61) | <0.01 |
| PPI/PCAB use | +2.18 | 8.81 (1.90-42.8) | <0.01 |
| CRP≥0.3 mg/dL | +0.04 | 1.04 (0.36-2.70) | 0.94 |

Odds ratios (OR) were derived by exponentiating the penalized coefficients. 5-ASA, 5-aminosalicylic acid; PPI, proton pump inhibitor; PCAB, potassium competitive acid blocker; CAI, C-reactive protein.

Supplementary Table 3. Characteristics of patients treated with topical 5-ASA only

| **Variable** | **n (%)** |
| --- | --- |
| Formulation  Enema  Suppository | 3 (16.7)  15 (83.3) |
| PPI/PCAB use | 1 (5.6) |
| 5-ASA Intolerance | 1 (5.6) |

5-ASA, 5-aminosalicylic acid; PPI, proton pump inhibitor; PCAB, potassium competitive acid blocker.
