# Supplementary figures and images for: Association of Proton Pump Inhibitor and Potassium‐Competitive Acid Blocker Use With Discontinuation and Intolerance of Oral 5‐Aminosalicylic Acid in Patients With Ulcerative Colitis
Source: JGH Open. 2026 Jan 31;10(2):e70350. doi: 10.1002/jgh3.70350 (PMC12860887; doi:10.1002/jgh3.70350)

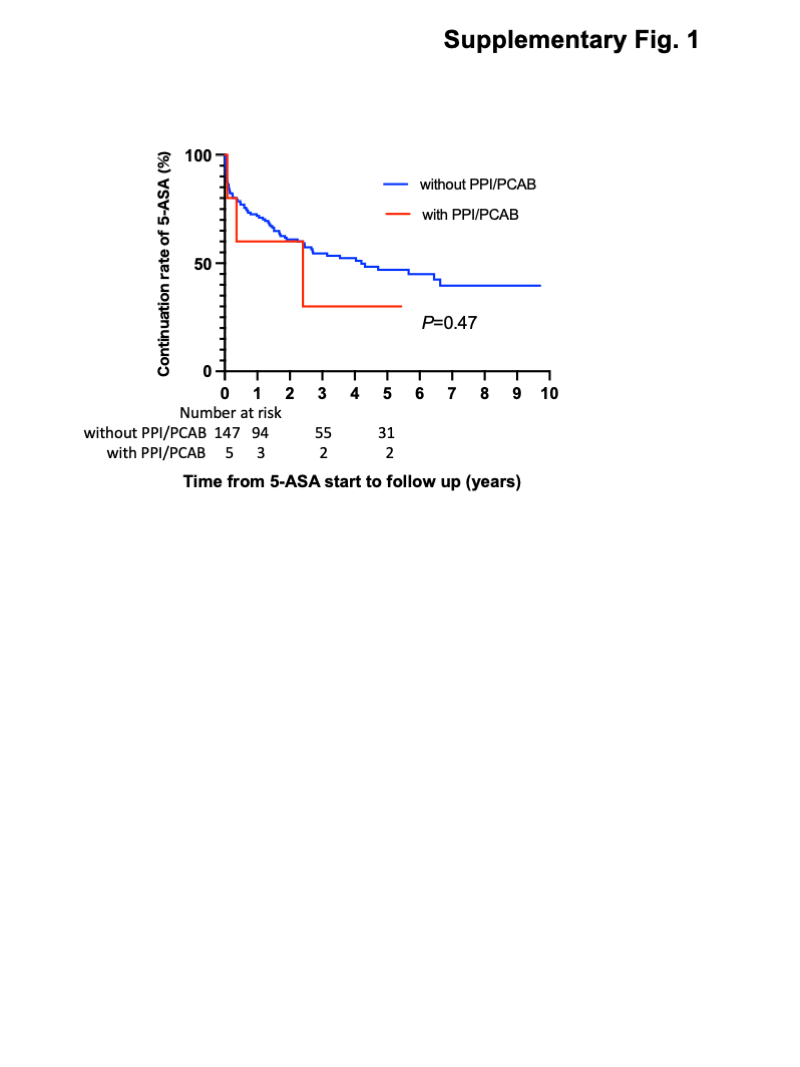

Supplement: Supplementary file 2 — Figure S1: Kaplan–Meier curve of 5‐ASA continuation excluding intolerance‐related discontinuations. p value was determined using a log‐rank test. 5‐ASA, 5‐aminosalicylic acid; PCAB, potassium‐competitive acid blocker; PPI, proton pump inhibitor. [file JGH3-10-e70350-s003.tiff]

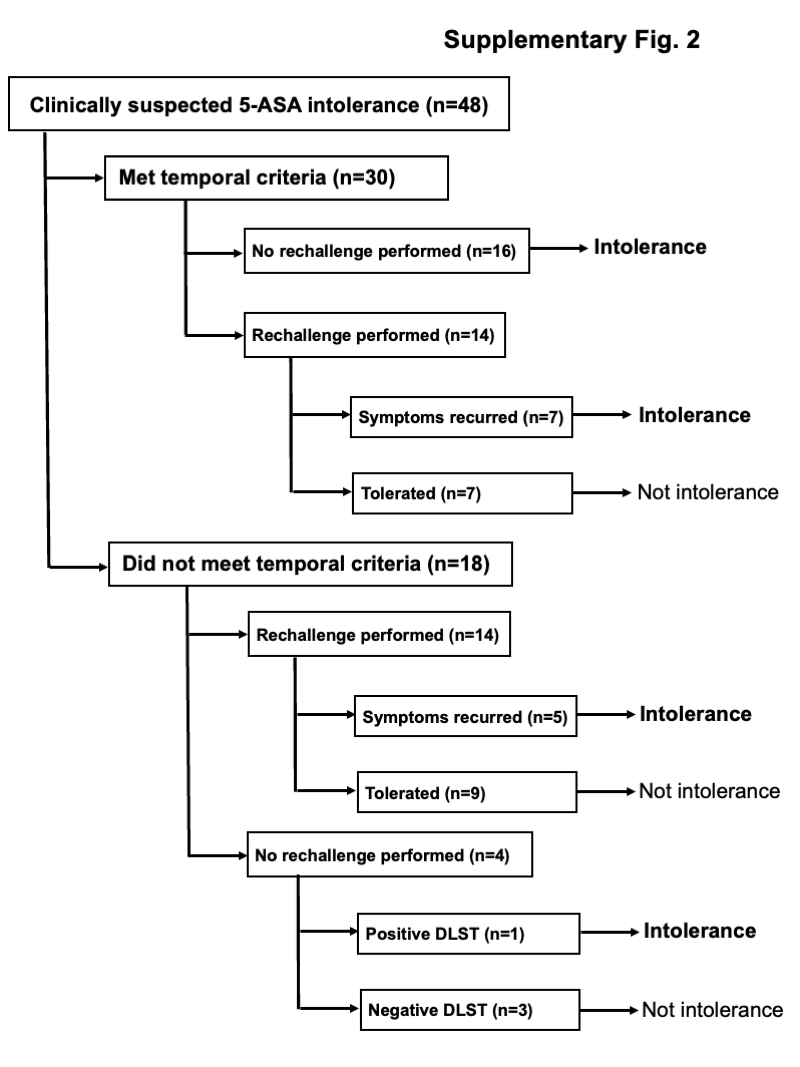

Supplement: Supplementary file 3 — Figure S2: Diagnostic flow of 5‐ASA intolerance. ASA, 5‐aminosalicylic acid; DLST, drug lymphocyte stimulation test. [file JGH3-10-e70350-s001.tiff]
